# Two Cases of Small Cell Cancer of the Maxillary Sinus Treated with Cisplatin plus Irinotecan and Radiotherapy

**DOI:** 10.1155/2013/893638

**Published:** 2013-01-29

**Authors:** Kiyoaki Tsukahara, Kazuhiro Nakamura, Ray Motohashi, Hiroki Sato

**Affiliations:** Department of Head and Neck Surgery, Tokyo Medical University Hachioji Medical Center, 163 Tate-machi, Hachioji, Tokyo 193-0998, Japan

## Abstract

*Background*. Small cell carcinoma (SmCC) in the nasal cavity and paranasal sinuses is very rare, and definitive therapies have not yet been established. *Methods*. Chemoradiotherapy comprised 60 Gy of external radiation, with the administration of irinotecan intravenously at 60 mg/m^2^ on days 1, 8, and 15 and cisplatin at 60 mg/m^2^ on day 1. *Results*. Case 1 involved a 66-year-old woman with stage III cancer. Adverse events included decreased white blood cells, anemia, and oral mucositis, all Grade 2. The patient remained free of cancer as of 3 years and 6 months after completing the treatment. Case 2 involved a 60-year-old man with stage IV cancer. He also experienced adverse events of decreased white blood cells, anemia, and oral mucositis, all Grade 2. He died after 11 months due to metastases throughout the body. *Conclusions*. The results suggest that this regimen may be tolerable as a therapy for this type of carcinoma.

## 1. Background

Extrapulmonary small cell carcinoma (SmCC) accounts for only 0.1% to 0.4% of all SmCC cases [1]. As SmCC of the head and neck progresses very rapidly and distant metastases occur readily, the prognosis is very poor [2]. In the field of head and neck cancer, however, the development of SmCC in the nasal cavity and paranasal sinuses is very rare. In the English literature, only 40 cases of SmCC of nasal cavity and paranasal sinuses have been reported [3]. As a result, definitive therapies have yet to be established for SmCC in this area. A phase II study and a multicenter, randomized phase III study reported that irinotecan (CPT-11) plus cisplatin (CDDP) were useful against SmCC of the lung [4, 5]. Noda et al. compared CPT-11 plus CDDP with etoposide (VP-16) plus CDDP in patients with extensive (metastatic) small-cell lung cancer. The median survival was 12.8 months in the CPT-11-plus-CDDP group and 9.4 months in the VP-16-plus-CDDP group (*P* = 0.002 by the unadjusted log-rank test). At two years, the proportion of patients surviving was 19.5 percent in the CPT-11-plus-CDDP group and 5.2 percent in the VP-16-plus-CDDP group [5]. Hakuba et al. reported the case report of CPT-11 plus CDDP in a chemoradiotherapy (CRT) regimen for SmCC of the nasal cavity [3]. We therefore used CPT-11 plus CDDP in a (CRT) regimen for SmCC of the maxillary sinus.

We report two cases of small cell carcinoma of the maxillary sinus, a very uncommon cancer, in which CDDP, CPT-11, and radiotherapy were combined for tolerable treatment. The purpose of the present paper is to report on the feasibility of this regimen as well as its tolerableness.

## 2. Methods

**Figure 1:**
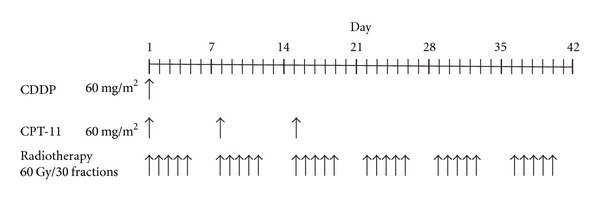
Regimen of irinotecan plus cisplatin in chemoradiotherapy.

CRT comprised 60 Gy of external radiation, with administration of CPT-11 intravenously at 60 mg/m^2^ on days 1, 8, and 15 and CDDP at 60 mg/m^2^ on day 1 (Figure 1). IRB of our hospital approved this CRT.

## 3. Case Report

**Case 1**

**Figure 2:**
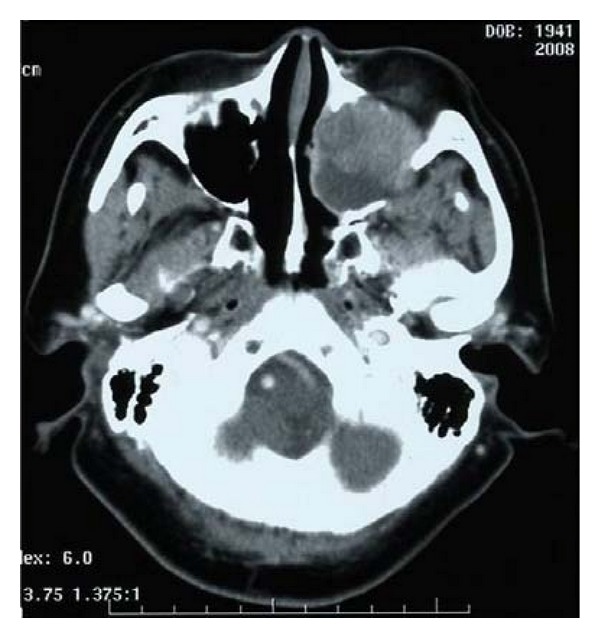
Tumor centered on the maxillary sinus with infiltration of the left orbit.

**Figure 3:**
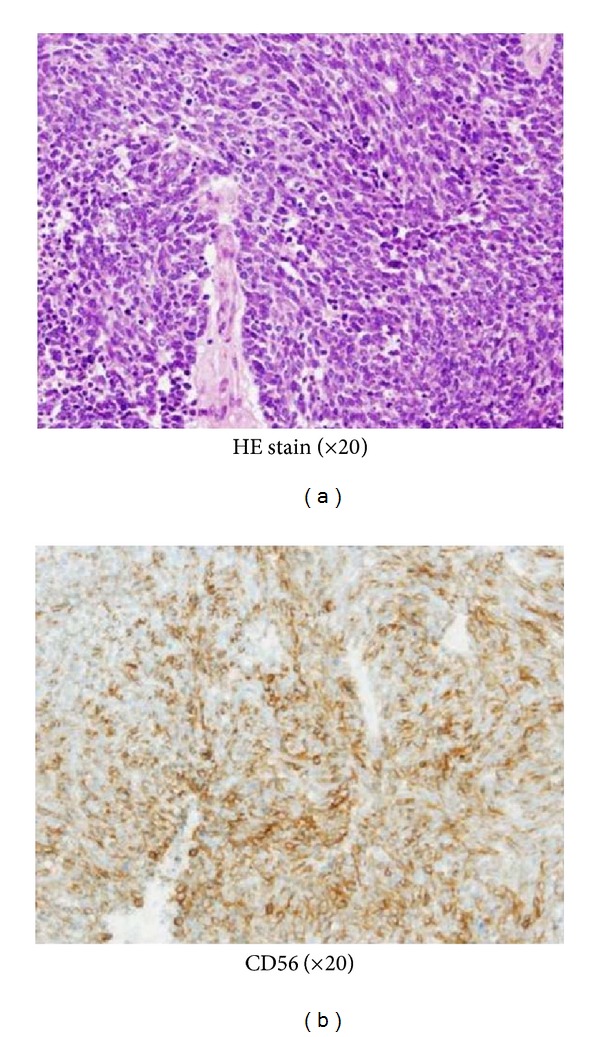
(a) Infiltrative, proliferative tumor that had formed various large and small solid cancer nests. (b) Results of CD56 staining were positive.

A 66-year-old Japanese woman visited our hospital with a 1-month history of nasal bleeding. Computed tomography (CT) of the neck revealed a tumor centered on the maxillary sinus and infiltration of the left orbit was observed (Figure 2). Caldwell-Luc antrostomy was performed. Pathologically, an infiltrative, proliferative tumor that had formed various large and small solid cancer nests was identified. Immunostaining showed CK (+), CD56 (+), CG-A (+), TTF-1 (+), and p63 (−). The Ki-67 index was high, as 40% to 90%, and SmCC was diagnosed (Figure 3). On the basis of positron-emission-tomography- (PE-) CT and chest CT, the tumor was determined to be T3N0M0 stage III.

**Figure 4:**
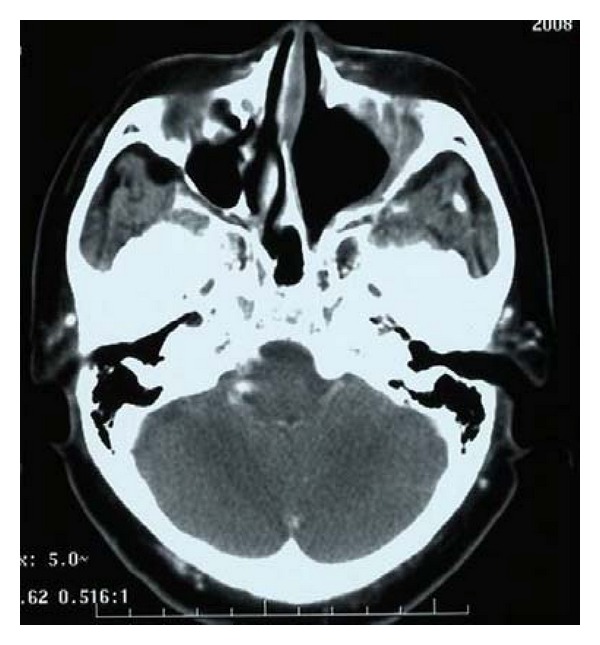
Soft tissue shadows remained. No cancer cells were observed in biopsy samples from three locations.

CRT including CPT-11 and CDDP was initiated. Adverse events included leukopenia, anemia, and mucositis, all of Grade 2. No increase in creatinine levels, diarrhea, or thrombocytopenia was seen. CT after the first month of treatment showed that while soft tissue shadows remained (Figure 4), no cancer cells were apparent in biopsy samples from three locations. Considering the soft tissue shadows to represent a postoperative change, clinical response was evaluated as a complete response. The patient subsequently completed four more courses of chemotherapy using CPT-11 and CDDP, but administration was then suspended in accordance with the wishes of the patient. Since then, the patient has been followed up with treatment. As of 3 years and 6 months after completing the last course of chemotherapy, no recurrences or metastases have been identified.

**Case 2**

**Figure 5:**
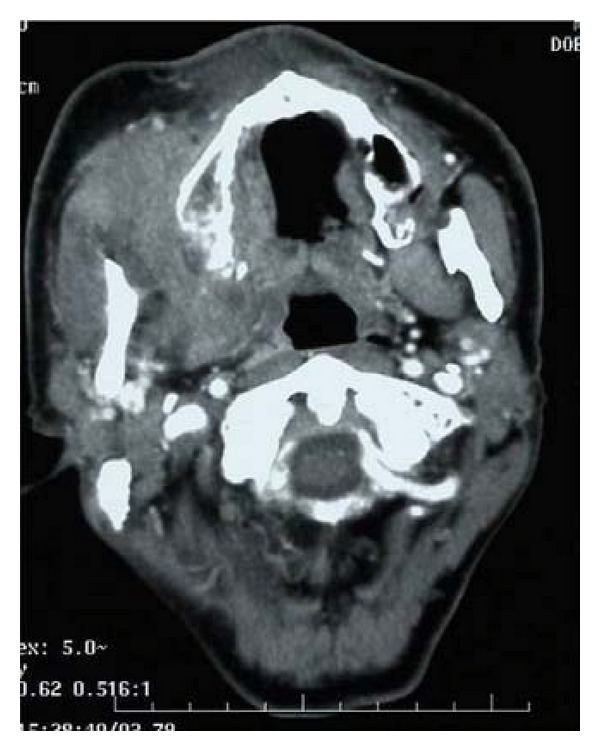
Tumor centered on the right maxillary sinus infiltrated the subcutis, right orbit, pterygopalatine fossa and ethmoid sinus.

**Figure 6:**
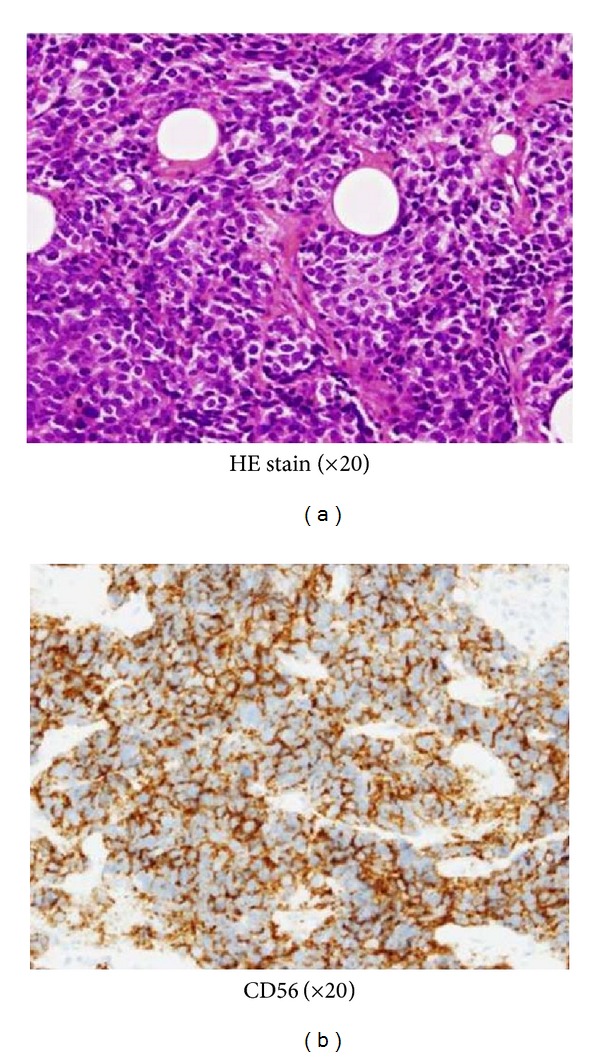
(a) The tumor showed a solid medullary growth pattern consisting of small-sized epithelial dysplasia. (b) Results of CD56 staining were positive.

Case 2 involved a 60-year-old man who visited our hospital with a chief complaint of cheek pain that had persisted for 2 months. Neck CT revealed a tumor centered on the right maxillary sinus infiltrating the subcutis, right orbit, pterygopalatine fossa, and ethmoid sinus (Figure 5). Biopsies were obtained under local anesthesia. Pathologically, the tumor showed with a solid medullary growth pattern consisting of small-sized epithelial dysplasia. Immunostaining revealed CD56 (+), CG-A (+), TTF-1 (−), and MNF-116 (+). The Ki-67 index was 80%, and SmCC was diagnosed (Figure 6). On the basis of PET-CT and chest CT, the tumor was determined to represent T4aN1M0 stage IVA.

**Figure 7:**
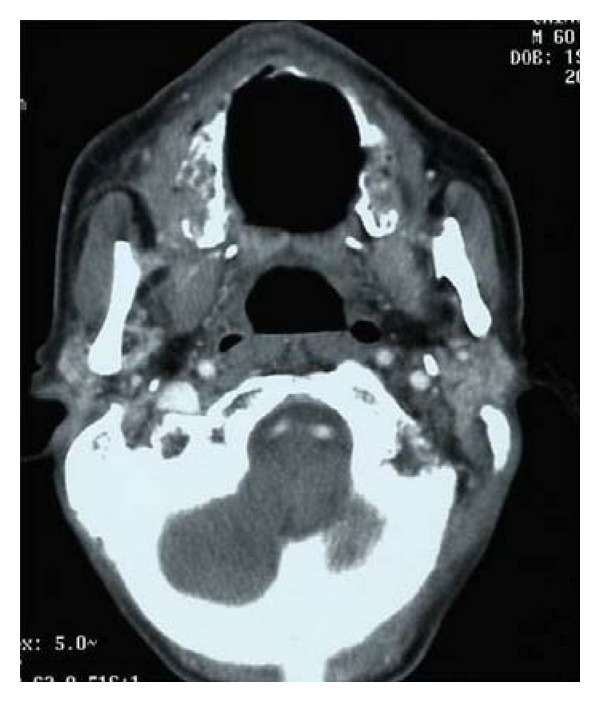
Despite a decrease in tumor size, soft tissue shadows remained and the evaluation was clinical PR.

CPT-11 and CDDP were administered in the CRT. Adverse effects of leukopenia, anemia, diarrhea, and mucositis were observed, all of Grade 2. In addition, Grade 1 thrombocytopenia was seen. No increase in creatinine levels was identified. CT in the first month after finishing the treatment showed that despite a decrease in tumor size, soft tissue shadows remained. The clinical outcome was thus evaluated as partial response (PR) (Figure 7). The patient subsequently received two further courses of chemotherapy using CPT-11 and CDDP, but multiple metastases to the lungs and liver were identified in the 5th month after finishing CRT. Although chemotherapy was changed to CDDP and etoposide, in the 7th month after finishing chemoradiotherapy, metastases to the lumbar vertebrae were identified, and radiotherapy at 30 Gy was performed. In the 11th month after finishing chemoradiotherapy, metastasis to the scalp was found, along with the enhancement of multiple metastases to the lungs and liver. The patient died in the 12th month after finishing CRT.

## 4. Discussion

Kudoh et al. evaluated the efficacy and toxicity of CPT-11 combined with CDDP in a phase II study of patients with previously untreated small-cell lung cancer [4], finding an overall response rate of 84%. Noda et al. conducted a multicenter, randomized phase III study that compared CPT-11 plus CDDP to VP-16 plus CDDP in patients with extensive metastatic SmCC [5]. They concluded that CPT-11 plus CDDP offered an effective treatment for metastatic small-cell lung cancer. Another study achieved a long survival period for SmCC of the nasal cavity using CPT-11 plus CDDP [6]. On the other hand, Kane et al. demonstrated that radiotherapy was effective for SmCC [7]. In view of these findings, we thought that systemic therapy using CPT-11 plus CDDP in conjunction with radiotherapy would prove effective against SmCC of the maxillary sinus. No complications of Grade 3 or over were seen in the 2 patients who received this therapy, suggesting that it might represent an acceptable regimen. However, various complications of Grade 3 and above have been reported for chemotherapy consisting of CPT-11 and CDDP, including leukopenia in 45%, neutropenia in 77% diarrhea in 19%, and anemia in 39% [4]. While few cases of this type of cancer have been encountered, the adverse events should be studied in the future.

 Regarding efficacy, Case 1 remained free of recurrences or metastases for at least 3 years and 6 months, indicating that this regimen suppressed the cancer locally. However, local suppression was not clear in Case 2 as the post-CRT evaluation was also PR.Potential cause for the differences in success is that Case 2 was more advanced disease.

 Although only 2 patients were treated and lesions were suppressed in only one, our findings still suggest that this regimen may be tolerable as a therapy for SmCC of the maxillary sinus.
